# Frailty and cognitive performance of elderly in the context of social vulnerability

**DOI:** 10.1590/1980-57642018dn12-020010

**Published:** 2018

**Authors:** Isabela Thaís Machado de Jesus, Fabiana de Sousa Orlando, Marisa Silvana Zazzetta

**Affiliations:** 1 Gerontóloga, Doutoranda pelo Programa de Pós-Graduação em Enfermagem da Universidade Federal de São Carlos, SP, Brazil.; 2 Assistente Social, Cargo de Professor Adjunto II pelo Departamento de Gerontologia da Universidade Federal de São Carlos, São Carlos, SP, Brazil.; 3 Enfermeira, cargo de Professor Adjunto II pelo Departamento de Gerontologia da Universidade Federal de São Carlos, São Carlos, SP, Brazil.

**Keywords:** frail elderly, cognition, social vulnerability, primary care, idoso fragilizado, cognição, vulnerabilidade social, atendimento primário

## Abstract

Elderly who live in the context of social vulnerability have lower education and socioeconomic status.

**Objective:**

To analyze cognitive performance as a factor associated with frailty status in elderly living in contexts of social vulnerability.

**Methods:**

An exploratory, comparative, cross-sectional study using a quantitative method was conducted with elderly people registered at Social Assistance Reference Centers. A semi-structured interview, the Edmonton Frail Scale and Montreal Cognitive Assessment were applied. The project was approved by the Research Ethics Committee. To analyze the data, a logistic regression was performed considering two groups (frail and non-frail).

**Results:**

247 elderly individuals participated in the study, with a mean age of 68.52 (±SD =7.28) years and education of 1-4 years (n=133). All the elderly evaluated resided in vulnerable regions. Regarding frailty, 91 (36.8%) showed frailty at some level (mild, moderate or severe) and 216 (87.4%) had cognitive impairment. On the regression analysis, frailty was associated with number of diseases (OR:1.60; 95%CI: 1.28-1.99) and cognition (OR:0.93; 95%CI: 0.89-0.98).

**Conclusion:**

Identifying level of frailty and cognition in socially vulnerable elderly reinforces the need for early detection in both these conditions by the public services that provide care for this population with a focus on prevention.

## Resumo

Idosos que vivem em contexto de vulnerabilidade social apresentam menor escolaridade e condição socioeconômica.

**Objetivo:**

Analisar o desempenho cognitivo como um fator associado para nível de fragilidade em idosos residentes em contextos de vulnerabilidade social.

**Métodos:**

Estudo exploratório, comparativo e transversal, com a utilização do método quantitativo realizado com idosos cadastrados em Centros de Referência de Assistência Social. Utilizou-seentrevista semi-estruturada, Escala de Fragilidade de Edmonton e Montreal Cognitive Assessment. O projeto foi aprovado pelo Comitê de Ética em Pesquisa. Para análise dos dados foi realizada regressão logística, considerando dois grupos (frágeis e não frágeis).

**Resultados:**

Participaram do estudo 247 idosos, com média de idade de 68,52 (dp=7,28) anos e com escolaridade de um a quatro anos (n=133). Todos os idosos avaliados residiam em regiões com vulnerabilidade. Quanto à fragilidade, 91 (36,8%) apresentaram fragilidade em algum nível (leve, moderada ou severa) e 216 (87,4%) apresentaram comprometimento cognitivo. Na análise de regressão, houve associação da fragilidade com número de doenças (OR:1.60; 95%IC: 1.28-1.99) e cognição (OR:0.93; 95%IC: 0.89-0.98).

**Conclusão:**

Identificar o nível de fragilidade e cognição de idosos em vulnerabilidade social reforça a necessidade de detecção precoce em ambas as condições por parte dos serviços públicos que assistem essa população com foco na prevenção.

Elderly who live in a context of social vulnerability have lower education and socioeconomic status.^1^ Social context is considered a risk factor for the development of cognitive impairment and consequently leads to the outcome of frailty.^2^ The social context, characterized by low financial means, income, education, working conditions, family structure, availability of services, sanitation, disease exposure and lack of social support networks, can contribute to the development of frailty.^3^

Frailty is defined as a dynamic state that affects an individual experiencing losses in one or more areas of human functioning, albeit physical, cognitive, social, problems with memory and attention, or reduced vision or hearing, and is caused by the influence of a range of variables that increase the risk of these outcomes, such as dependency, institutionalization and death.^4^^-^ 6 These changes reflect aging-related alterations and involve intrinsic and extrinsic factors which are part of a dynamic and complex system.^7^

Searle and Rockwood (2015) argued that frailty is a state of increased risk due to the accumulation of deficits and that this risk is related to the number of health deficits that an individual accumulates, and represents a strong risk factor for cognitive impairment.^8^ It is understood that frailty and cognition are linked and have implications. Although many of the instruments for measuring frailty do not assess cognition, it is evident that cognitive impairment is a characteristic of frailty.^9^ A study involving 304 elderly in a context of high social vulnerability in São Paulo, Brazil, showed that 27.3% of the elderly evaluated were frail (according to Fried’s phenotype) had a low educational level(mean 2 years) and presented cognitive impairment (41%).^10^ Rodriguez-Mañas et al.^11^ showed the trajectory of frailty that begins with cognitive decline may differ to the trajectory of frailty beginning with physical components.

In contexts of social vulnerability, characterized by socioeconomic and demographic dimensions, frailty can be influenced by (besides clinical issues) social exclusion, less access to information, social inequality and lack of social support. Similarly, cognition can be reduced as a result of behavioral issues, unhealthy living habits and lifestyles, educational level and low physical activity.^12^^,^ 13

Based on international consensus, some studies have considered cognition in the definition of frailty as “cognitive frailty”.^14^ Cognitive frailty is defined as a clinical manifestation characterized by the simultaneous presence of heterogeneous physical frailty and cognitive impairment.^13^ An international study found the presence of cognitive impairment in 40% of frail elderly. The authors also argued that cognition is one of the most rapidly deteriorating systems among the frail.^15^

Studies in the area of social vulnerability gain particular relevance when the social context is considered a risk factor for the development of cognitive impairment and consequently leads to the outcome of frailty. The literature still has gaps with regard to studies investigating frailty using the Edmonton Frail Scale and cognition using the Montreal Cognitive Assessment. In this perspective, evaluating elderly and identifying risk factors for frailty can result in prevention and consequently lower costs for health services, since in this context low education and income are determinants of adverse outcomes, related to less access to stimuli, restriction of external relationships and development of inefficacy. However, it is known that not all people with cognitive impairment become frail. The causes of frailty are unclear, from risk factors for frailty to predisposing factors for cognitive impairment, the hypothesis holds there may be an association between both. The aim of this study was to analyze cognitive performance as a factor associated with frailty status in elderly living in contexts of social vulnerability.

## Methods

This is an exploratory, comparative and cross-sectional study, based on the quantitative method of research, conducted in elderly people registered at five Social Assistance Reference Centers (CRAS) of the city of São Carlos, SP, located in vulnerable regions.

São Carlos city has 243,765 inhabitants and elderly correspond to 14.9% of the total population. The city has a monthly per capita income of US$ 232.35 and a Human Development Index (HDI) of 0.805. To cater for the population of São Carlos, this large city has five CRAS. Each CRAS caters for a maximum of 5,000 families, distributed across the reference sectors.^16^ The five CRAS have been denoted as I, II, III, IV and V. CRAS I, II and III are located in a region of high social vulnerability. CRAS IV covers regions with average vulnerability and CRAS V is located in a region with very low vulnerability. The social vulnerability of the region in which these elderly reside is classified as 2 on the São Paulo Social Vulnerability Index (IPVS). The IPVS classifies census sectors of the State of São Paulo in Brazil according to levels of vulnerability based on socioeconomic and demographic dimensions. The socioeconomic dimension includes: percentage of illiterate head of households; percentage of head of household with complete elementary education; average years of education of head of household; nominal household income; percentage head of household earning an income of up to 3 minimum wages (approximately US$ 920). The demographic dimension includes: percentage head of households with children >10 years; mean children per household; percentage with children aged 0 to 4 years.^17^

First we conducted a survey of all physical records of registered users at the CRAS, totaling 1451 elderly registered. After access to the registration name, age and address, an active search was performed on all addresses. A total of 1204 users were excluded on the grounds that 679 (46.7%) were not found at the registration address or addresses had changed or the user lived in areas outside the catchment area of the CRAS, and 439 (57%) corresponded to losses due to death, withdrawal or refusal, because the elder was alone or lacked understanding to answer the questions. Another 86 corresponded to caregivers of elderly people who were unable to answer the questions of the survey and were not alone in their homes. Therefore, this study included a final total of 247 interviews with elderly. All interviews took place at the residence of the elderly and the visit to households was made between Monday and Friday during business hours. The interview lasted approximately 45 minutes and was carried out by three previously trained undergraduate students on the Gerontology Course of the Federal University of São Carlos (UFSCar) to standardize the data collected. Data collection occurred from August 2012 to August 2016.

The sample was composed of 247 elderly registered at CRAS who met the following inclusion criteria: 60 years of age or older, registered at one of the CRAS, understood the questions in the interview, agreed to participate and sign the Free and Informed Consent Form. Exclusion criteria were: hearing or visual deficits that would prevent full understanding of the study.

A demographic questionnaire, a scale to determine frailty and a cognitive screening instrument were applied. The demographic questionnaire was previously devised by researchers and collected information on: gender (male and female), age (60-69, 70-79, 80-89 and more than 90 years), ethnicity (white, black, brown and yellow), marital status (married, single, widower, separated and divorced), religion (catholic, gospel, others or none), current occupation (retirees and non-retirees), time of retirement, education (illiterate, literate with no education, 1-4 years, 5-8 and ≥9 years) and number of reported diseases (none, 1-2 illness and more of 3). To identify frailty, the Edmonton Frailty Scale (EFS) was used, developed by Rolfson et al. in 2006 and translated and validated in Brazil by Fabrício-Wehbe in 2009. The EFS evaluates nine domains: cognition, general health status, functional independence, social support, medication use, nutrition, mood, continence and functional performance, comprising 11 items. The maximum score is 17 points, representing the highest level of frailty. Individuals scoring from zero to four points are considered “Non-Frail”, five to six “Apparently Vulnerable”, seven to eight points “Mild Frailty”, nine to 10 “Moderate Frailty” and 11 points or more “Severe Frailty.”^18^^,^ 19 Cognitive evaluation was performed by applying The Montreal Cognitive Assessment (MoCA) developed by Nasreddine in 2005 and translated and validated in Brazil by Bertolucci in 2008. The test evaluates eight areas: visuo-spatial abilities, executive functions, verbal-abstraction task, working memory, attention, concentration, serial subtraction task, digits forward and backward. The total score for the instrument is 30 points, and the cut-off 26 points. One point is added to the final score of individuals with ≤12 years of education. Participants that had a score below the cut-off of 26 points were considered as having cognitive impairment and above the cut-off of 26 no cognitive impairment.^20^^,^ 21 The data was stored using Microsoft Office Excel software (2010). Data analysis was performed on the Windows System, version 9.2 and treated with descriptive statistics. Due to the absence of normal distribution of the variables, nonparametric tests were applied. Cronbach’s alpha coefficient was used to verify internal consistency, where values >0.70 indicate high consistency. In this study, values of 0.530 and 0.835 were obtained for the EFS and MoCA, respectively. Due to the absence of normal variable distribution, Fisher’s Exact Test was used to compare categorical variables. Multivariate regression was used to verify the association of frailty with cognition. Univariate binary logistic regression was conducted, in which the presence of frailty according to the EFS was the dependent variable considering two groups, frail and non-frail. Independent variables analyzed had as categorical variables: gender, ethnicity, religion, marital status, education, reported illnesses and social vulnerability; and, as continuous variables: age, years of retirement, number of diseases, total score on MoCA and years of education. Of these variables analyzed in the univariate regression model. independent variables that had a p-value ≤0.2 were included in the multivariate model. The independent variables with p-value ≤0.05, adjusted for sex, age and education, were retained in the multivariate model.

All ethical principles were observed, as per Resolution 466/12 of the Conselho Nacional de Saúde. This study used and expanded the research database entitled: “The frailty of the elderly and the Basic System of Social Assistance” with the approval of the Research Ethics Committee of the Federal University of São Carlos (under permit: 72182/2012). This research was approved under permit: 1785874, on October 21, 2016, CAAE: 57857016.0.0000.5504.

## Results

The study sample comprised 247 elderly, registered at five CRAS in São Carlos. There was a predominance of individuals that were female (79.8%), aged 60 to 69 years (64.8%), white (57.5%), married (44.1%) and of the catholic religion (61.1%). Most participants were retired (55.5%) and had from one to four years of education (53.9%). Mean participant age was 68.5 years (±SD=7.3) and retirement time was 11.5 years (±SD=13.3). In relation to health, 53.8% of the elderly reported two diseases. Regarding the vulnerability of the region in which the participants lived, most resided in regions of high social vulnerability (58.3%). It was found that 41.7% of elderly assessed did not exhibit frailty whereas 36.8% had some degree (mild, moderate or severe) of frailty and 216 (87.4%) had cognitive impairment. The distribution of sociodemographic characteristics, vulnerability and frailty and cognition data of the interviewed elderly are given in Table 1.

**Table 1:** Distribution of sociodemographic characteristics, vulnerability, frailty and cognition of elderly registered at CRAS. São Carlos, SP, 2016 (n=247).

Variables	Categories	N (%)	Mean ( ±SD)	[Min-Max]	Median
Gender	Female	197 (79.8)			
	Male	50 (20.2)			
Age range	60-69 years	160 (64.8)			
	70-79 years	64 (25.9)			
	80-89 years	19 (7.7)			
	≥90 years	4 (1.6)			
Age (in years)		247	68.5 (7.3)	[60-94]	66
Ethnicity	White	142 (57.5)			
	Black	69 (27.9)			
	Brown	35 (14.2)			
	Yellow	1 (0.4)			
Marital status	Married	109 (44.1)			
	Single	6 (2.4)			
	Widower	94 (38.1)			
	Separated	20 (8.1)			
	Divorced	18 (7.3)			
Religion	Catholic	151 (61.1)			
	Gospel	74 (30.0)			
	Others	16 (6.4)			
	None	6 (2.5)			
Current occupation	Retirees	137 (55.5)			
	Non-retirees	110 (44.5)			
Time of retirement (in years)		137	11.5 (13.3)	[0-63]	8
Education	Illiterate	45 (18.2)			
	Illiterate with no education	23 (9.3)			
	1-4 years	133 (53.9)			
	5-8 years	35 (14.2)			
	≥ 9 years	11 (4.4)			
Reported diseases	None	14 (5.7)			
	1-2 diseases	133 (53.8)			
	≥ 3 diseases	100 (40.5)			
Number reporting diseases		247	2.4 (1.5)	[0-7]	2
Social vulnerability	High (CRAS I,II,III)	144 (58.3)			
	Average (CRAS IV)	56 (22.7)			
	Very low (CRAS V)	47(19.0)			
	Non-frail	103 (41.7)			
	Apparently vulnerable	53 (21.5)			
Frailty level	Mild frailty	50 (20.2)			
	Moderate frailty	30 (12.1)			
	Severe frailty	11 (4.5)			
Cognitive Impairment	Impairment	216 (87.4)			
	No impairment	31 (12.6)			

SD: standard deviation; Min: minimum value; Max: maximum value.

On the assessment of level of frailty, 216 (87.44%) participants scored below the cut-off of 26 points and 84 (38.88%) had some level of frailty (mild, moderate or severe), as shown in Table 2.

**Table 2:** Comparison of level of frailty according to the EFS for cut-off score on the MoCA of elderly registered at CRAS. São Carlos, SP, 2016 (n=247).

Cut-off score	Total	Non-Frail	Vulnerable	Mild	Moderate	Severe
<26	**216**	81 78.64%	51 **96.23%**	46 **92%**	28 **93.33%**	10 **90.91%**
≥ 26	**31**	22 **21.36%**	2 3.77%	4 8%	2 6.67%	1 9.09%
	**Total**	103	53	50	30	11

p-value=0.011

Factors associated with frailty included number of diseases (OR:1.60; 95%CI: 1.28-1.99) and cognition (OR:0.93; 95%CI: 0.89-0.98), as shown in the Table 3.

**Table 3:** Variables associated with the condition of frailty of elderly registered at CRAS. São Carlos, SP, 2016 (n=247).

					95%CI	
Variables	Category	B	p-value	OR	Lower	Upper
Number reporting diseases		0.47	<0.001	1.60	1.28	1.99
MoCA Total		–0.06	0.005	0.93	0.89	0.98
Age (years)		0.03	0.15	1.03	0.98	1.08
Gender	Female	–0.10	0.79	0.90	0.42	1.93
Education (years)		–0.14	0.34	0.86	0.63	1.17

## Discussion

In the present study, there was a predominance of participants who were female, with a mean age of 68.5 years, white, married, a low educational level and retirees, similar to data for research involving elders from the community in the national context.^21^^-^ 25 The multivariate regression of the variables in this study was statistically significant for the number of diseases and cognition, in accordance with other studies in vulnerable elderly populations.^8^^,^ 10 ^,^ 26 The data obtained indicate a demographic prevalence of females, which corroborates the concept of feminization in old age. In fact, women have higher life expectancy, lower rates of mortality from external causes, less exposure to occupational hazards, and consume less tobacco and alcohol compared to men, and more frequently seek health and social services.^27^

The low level of education found in this study may be a result of living conditions. Evidence indicates that level of education is a protective factor for the adverse effects to health in older persons.^2^ In addition, elderly people with low education may have mental health problems, chronic conditions, as well as social exclusion, less access to information and unfavorable socioeconomic conditions.^12^ The literature shows that education can be regarded as a protective factor against cognitive impairment in aging. A multicenter study conducted in the community with Brazilian elderly identified cognitive deficit in 24.8% of the study population and educational level of one to four years.^28^ Cognitive impairment in the elderly with lower educational level may be related to less access to stimuli, restricted external relationships and the development of a feeling of ineffectiveness over the years, resulting in frailty.^29^^,^ 30

Regarding current occupation, in this study there was predominance of elderly retirees, accounting for 137 (55.4%) of the 247 interviewed. Andrew (2010) presented socioeconomic status in the elderly as a broad concept that includes factors such as education, occupation, income, wealth and deprivation.^31^ In most cases, income affects the health of those that have limited access to services. Another view is that education influences health through lifestyle and behaviors.^32^

Regarding vulnerability of social context, 58.3% of respondents resided in regions with high vulnerability. Armstrong et al. (2015) conducted a study to determine the association of vulnerability with cognition in elderly Japanese and found that greater social vulnerability was associated with a greater chance of cognitive decline.^33^

Of the 247 elderly interviewed, 36.8% had some level of frailty - mild, moderate or severe - similar to data found in the literature. A study of elderly in primary care from the interior of São Paulo interviewed 128 elderly and found that 21.4% were vulnerable and 30.1% showed some level of frailty, according to the EFS.^35^ Another study conducted with 240 community elderly in the interior of São Paulo found that 39.1% were frail.^36^ Over the past five years, evaluating frailty in elderly has been of interest to researchers with the intention of checking those most in need of medical attention and assistance, with the aim of developing strategies for prevention in the context in which they live. Accordingly, identification and assistance for eradicating, preventing and delaying frailty, when possible, should be incorporated into services in research, with assessments centering on initial identification of the syndrome, focusing particularly on disadvantaged sectors with a view to improving the quality of life in old age.

The elderly respondents of this study presented possible cognitive impairment. Comparison of frailty with cognition revealed that 38.8% of frail elderly had cognitive impairment, which corroborates findings in the literature. A study in Brazilian elderly using other instruments to measure frailty and cognition found low cognitive performance for frail older adults aged 65 to 74 years and showed weakness.^37^ Another study conducted in elderly which used the EFS to determine frailty and another instrument to check cognition obtained from the frailty, showed that 20% had cognitive deficit.^24^

In this study, we found evidence that for every negative point for cognition there was a 0.93 greater chance of the person being frail. The literature shows that the presence of cognitive deficit causes difficulties in the elderly, in addition to changes in self-esteem and quality of life.^38^ In addition, elderly tend to experience a decrease in cognitive ability with aging, where this may be driven by genetic or cultural factors, life habits and presence of comorbidities.^34^ Given the heterogeneous nature of frailty and cognition - which is also linked to functional decline and capacity - constituting a component of frailty, research is needed to determine the factors that contribute to the frailty syndrome and special attention dedicated to those with cognitive impairment through evidence-based interventions in multiple domains.^13^^,^ 39 ^,^ 40

No consensus on the definition of frailty exists in the literature and there are numerous tools for evaluating frailty and for cognitive screening.^14^ While researchers, policy makers and health care providers generally agree that frailty may have a major impact on society, because it is at greater risk of adverse outcomes, frailty may be reversible with the redirection of actions on the part of primary care services. It is known that cognitive decline is a risk factor for frailty and can lead to falls, institutionalization, hospitalization and death. The identification of frail individuals can facilitate decision-making and management of adverse outcomes in both conditions.

The combination of frailty and cognition increases the risk of adverse health outcomes. The results of this study suggest that cognitive impairment may be a clinical feature of frailty or there may be common factors that predispose people to both outcomes, such as sociodemographic issues.^41^ The scientific literature suggests that frailty and cognition interact within a cycle of decline associated with aging, presenting immune dysfunction and neuroendocrine dysregulation.^42^ The literature shows significant associations between high levels of Interleukin 6 (IL-6) and high levels of frailty.^43^ At the same time, dysregulation of inflammatory pathways can affect the central nervous system and be involved in the pathophysiological mechanisms of neurodegenerative diseases, such as Alzheimer’s disease.^44^ According to Ruan et al. (2017), biomarkers can be useful for early detection, screening, diagnosis, and clinical research of these pathologies, which seek to determine pathogenic stage and whether the body has an effective response to the treatment to which it is being subjected.^45^

Researchers claim that there seems to be a biological association between cognitive impairment and frailty, and that both conditions may share the same pathophysiological mechanisms, although the underlying mechanisms remain unclear.^46^ A number of mechanisms have been proposed to explain the link between the relationship of frailty and cognitive impairment, which involve inflammatory processes, neuroendocrine dysregulation, oxidative stress, hormonal dysfunction, low Vitamin D, malnutrition and cardiovascular risks. Chronic inflammation plays a fundamental role in the pathogenesis of frailty and elevated levels of inflammatory cytokines are also associated with cognitive impairment through involvement in the disruption of brain mechanisms, hormone dysregulation and oxidative stress. The hormone testosterone is believed to have the function of a protective effect on cognition through synaptic plasticity in the hippocampus and controlling protein beta amyloid accumulation, while the hormone is associated with reduced muscle strength with age. Insulin is another proposed mediator between frailty and cognition, considering insulin resistance with age.^47^ Vitamin D, also has a correlation with frailty and cognition, with low levels of vitamin D indicating cognitive impairment, lower processing of the information and decreased physical functioning.^42^ Nutrition can present a link between cognition and frailty due to biological and behavioral effects and, cardiovascular risk may be another factor between frailty and cognition. It is known that impaired muscle function entails, specifically in cardiovascular diseases, small vessels and low-grade inflammation, impairing blood flow, causing sarcopenia and an increased risk of developing vascular dementia.^48^

Future studies should investigate frailty in relation to cognition in vulnerable settings, given their scarcity in the scientific literature, to inform public services for readjustment of actions and prevention. Consequently, the identification of people with frailty and cognitive impairment is essential for immediate interventions. In the context of social vulnerability, social risk factors may also affect health outcomes in the elderly. Canadian studies have shown that an overall measure of social vulnerability combining a variety of factors into a single measure can predict negative health outcomes including cognitive decline. Although social vulnerability may provide insights into adverse risks, it is still unclear how this impacts health and aging of the elderly.^49^

Limitations of this study include the cross-sectional design employed which does not allow causality to be established between the explanatory and outcome variables to formulate hypotheses. The sample size may limit the generalizability of the results, however, a large number of refusals can be expected in research using an active search of participants. There was difficulty accessing registered users of the CRAS on account of social issues: violence, trafficking in narcotics and unhealthiness of the residence for the elderly. This study made it possible to provide information about the frailty status in social vulnerability in relation to cognition. There was no indication that the elderly with some level of frailty showed cognitive impairment. Frailty was negatively associated with cognition and was statistically significant. To tackle frailty and track the cognition of vulnerable elderly requires early detection on the part of the public services caring for this population as a means of alerting to the risks. These results do not reduce the importance of conducting research in a context of social vulnerability, since the extrinsic context implies risks to the individual.
